# Coarse and Fine Two-Stage Calibration Method for Enhancing the Accuracy of Inverse Finite Element Method

**DOI:** 10.3390/s23135793

**Published:** 2023-06-21

**Authors:** Jiewei Lu, Dahang He, Zhenyi Zhao, Hong Bao

**Affiliations:** 1 Key Laboratory of Electronic Equipment Structure Design, Ministry of Education, Xidian University, Xi’an 710071, China; 21041110429@stu.xidian.edu.cn (J.L.); hdh@stu.xidian.edu.cn (D.H.); 22041212766@stu.xidian.edu.cn (Z.Z.); 2 Intelligent Robot Laboratory, Hangzhou Research Institute of Xidian University, Hangzhou 311231, China

**Keywords:** inverse finite element method, shape sensing, single-objective particle swarm optimization, error correction model, Bayesian, residual analysis, fuzzy network

## Abstract

The inverse finite element method (iFEM) is a novel method for reconstructing the full-field displacement of structures by discrete measurement strain. In practical engineering applications, the accuracy of iFEM is reduced due to the positional offset of strain sensors during installation and errors in structural installation. Therefore, a coarse and fine two-stage calibration (CFTSC) method is proposed to enhance the accuracy of the reconstruction of structures. Firstly, the coarse calibration is based on a single-objective particle swarm optimization algorithm (SOPSO) to optimize the displacement–strain transformation matrix related to the sensor position. Secondly, as selecting different training data can affect the training effect of self-constructed fuzzy networks (SCFN), this paper proposes to screen the appropriate training data based on residual analysis. Finally, the experiments of the wing-integrated antenna structure verify the efficiency of the method on the reconstruction accuracy of the structural body displacement field.

## 1. Introduction

Shape sensing is significant for the safety monitoring of structures such as aircraft and radar antennas. This technology is applied in various aspects of aerospace, military and civil applications [1,2,3]. Considering that the array accuracy and electrical performance are affected by antenna deformation [4,5,6], the shape-sensing technique is significant for antenna performance. The shape-sensing method can currently be divided into two categories [7,8]: one is non-contact measurement and the other is contact measurement. Non-contact measurement is based on laser tracking and the laser position sensor method to measure displacement directly, and real-time measurement is difficult to achieve due to the high requirements for the measurement environment and measurement instruments in practical engineering applications. The contact measurement method is used to calculate the deformation and displacement of the structure by using strain sensors to obtain strain information, which has promising applications in practical engineering [9,10,11].

The methods for reconstructing the structural displacement field based on strain information are divided into: KO displacement theory, modal method and iFEM. The Ko displacement theory establishes a mathematical model for the strain according to the primary or secondary distribution and combines it with the shape function to calculate the displacement [12,13]. However, this method is only applicable to the problem of reconstructing the unidirectional displacement field. The modal method is to measure the strain values and strain modes at specified locations, solve for the modal coordinates of each order mode, and then obtain the displacement field of the structure [14,15]. But this method requires a high-precision physical model and is not applicable to the deformation reconstruction of structures under a high-frequency excitation response. The iFEM was proposed by Tessler and Spangler [16,17], with the most widespread and promising applications among the three methods. This method discounts the material properties of the structure and the information of the applied load, with the advantages of high reconstruction accuracy and rapid reconstruction of the displacement field. The iFEM is based on the least-squares variation principle. The strain values are measured experimentally, and a mathematical model of strain–displacement is established. Kefal and Oterkus et al. [18] proposed an inverse finite element model with a quadrilateral cell. Compared to the triangular cell, the generation of singular matrices in the inverse operation is avoided due to the increase in angular degrees of freedom in the z-direction, preventing the shear self-locking phenomenon of the shell structure when reconstructing the deformation field. Gherlone et al. [19,20] proposed to paste fiber brag grating (FBG) sensors on the surface of a Timoshenko beam to obtain the shape sensing of the beam structure in transverse shear, tension, bending and torsional deformation. Cerracchio et al. [21] proposed to combine the original inverse finite element formulation with the refined Zigzag theory, which is applicable to the deformation monitoring of multi-layer composites and sandwich structures. Bao et al. [22] proposed an inverse finite element model using a univariate to optimize the number of sensors installed on the Timoshenko beam. Niu et al. [23] proposed the inverse scaled boundary element to model the Kirchhoff plate structure reconstruction, and the method achieves 3D deformation reconstruction through the strain information on the single-layer surface of the plate structure.

In practical engineering, measurement errors due to the strain transducer installation offset and structural body errors can reduce the reconstruction accuracy of the iFEM. To solve the problem, Rosso et al. [24] proposed an improved particle swarm optimization algorithm that introduces a new local search operator to help solve feasible regions of challenging optimization problems. Zhao [25,26] proposed a particle swarm optimization algorithm to determine the sensor layout locations. Pan [27] proposed a method to calibrate strains by mapping experiments using fuzzy networks to measure strain values. However, structural errors can greatly affect the calibration results of this method. Fu et al. [28] proposed a method that combines support vectors with fuzzy networks to correct strain errors. The drawback of this method is the lack of a large amount of experimental working condition data, which will have an impact on the training of the fuzzy network. To address the problem of the effect of a small sample size on reconstruction accuracy, Xu et al. [29] proposed a two-step calibration method, but this method is limited to the reconstruction displacement accuracy at the maximum deformation position, and the reconstruction accuracy at the rest of the positions is not as accurate. Li et al. [30] determined the layout location of sensors based on a multi-objective particle swarm optimization algorithm. The fuzzy network calibration method for the small sample problem was proposed by Li et al. [31], which improves the reconstructed accuracy of the whole displacement field effectively. However, when strain sensors are artificially installed to measure strain values, the offset in the installation position of the sensor increases the error in the reconstruction of the deformation field.

For improving the accuracy of the reconstructed displacement field, this paper proposes a CFTSC method. Firstly, the coarse calibration method is based on a single-objective particle swarm optimization algorithm to correct the error of the displacement–strain matrix due to the offset of the sensor position installation. After the coarse calibration, the fine calibration of the system error based on the self-constructed fuzzy network is continued to further improve the accuracy of the reconstructed displacement field. Secondly, since a suitable training set can improve the training effect of self-constructed fuzzy networks, this paper proposes to screen suitable training data based on residual analysis. All samples are fitted with NURBS curves to obtain the residual value of each sample point, and the samples are classified according to the residual value of each sample, from which suitable training data are screened.

The sections of this paper are described as follows. In Section 2, the theoretical framework of beam iFEM is described. In Section 3, the specific method of CFTSC is introduced. In Section 4, The calibration effect of the proposed CFTSC method on the displacement field is illustrated experimentally. Conclusions are given in Section 5.

## 2. The Framework of the Inverse Finite Element Method

The iFEM is based on the least squares function between the theoretical sectional strain *e*(*u*) and the actual sectional strain $e^{\varepsilon }$. It can be expressed as:


(1) $$\Gamma (u)=∥e(u)-e^{\varepsilon }{∥}^{2}$$

The displacement $d^{t}$ at any position of the section can be represented by the shape function *H*(*x*) and the nodal degrees of freedom (DOFs) $u_{e}$.

(2) $$d^{t}=H(x)u_{e}$$

The strain at any position of the cross-section can be obtained from Equation (3).

(3) $$e_{u}=M(x)u_{e}$$

where *M*(*x*) is the derivative of *H*(*x*), called the strain function matrix. By taking the derivative of u in Equation (1), the derivative function of u is made to be zero, so that the difference *Γ*(*u*) between the theoretical strain and the actual strain is minimized. Equation (4) is obtained.

(4) $$k^{e}u_{e}=f^{\mu }$$

where $k^{e}$ and $f^{\mu }$ are denoted as

(5) $$\begin{array}{l} k^{e}=\sum_{p=1}^{6}w_{p}k_{p}^{e},k_{p}^{e}=\frac{l}{n}\sum_{i=1}^{n}\left[M_{p}^{T}(x_{i})M_{p}(x_{i})\right]\\ f^{\mu }=\sum_{p=1}^{6}w_{p}f_{p}^{e},f_{p}^{e}=\frac{l}{n}\sum_{i=1}^{n}\left[M_{p}^{T}(x_{i})e_{p}^{\varepsilon }(x_{i})\right] \end{array}$$

where *l* denotes the unit length after reconstruction, *n* denotes the number of section strains on the neutral axis, $x_{i}$ denotes the position where the calculated section strains make, and $w_{p}$ denotes the weighting factor. 

**Figure 1:**
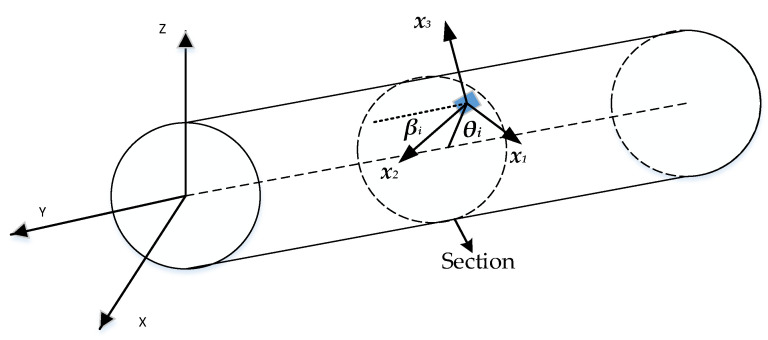
Installation position of the strain sensor on the outer surface of the beam.

When the strain sensor layout position is determined, the param*eters x = x_i_, θ = θ_i_, β = β_i_* (*i* = 1, 2, …, 6) related to the sensor position are also determined. The surface strains $\varepsilon^{a}$ are then calculated by Equation (6).

(6) $$\begin{array}{ll} \varepsilon^{a}(x_{i},\theta_{i},\beta_{i}) & =[(cos^{2}\beta -\mu sin^{2}\beta ),(cos^{2}\beta -\mu sin^{2}\beta )sin\theta \times r,(cos^{2}\beta -\mu sin^{2}\beta )cos\theta \times r,\\ & cos\beta sin\beta cos\theta ,cos\beta sin\beta sin\theta ,cos\beta sin\beta \times r]\\ & \times [e_{1}^{\varepsilon }(x_{i}),e_{2}^{\varepsilon }(x_{i}),e_{3}^{\varepsilon }(x_{i}),e_{4}^{\varepsilon }(x_{i}),e_{5}^{\varepsilon }(x_{i}),e_{6}^{\varepsilon }(x_{i}){]}^{T}\\ & =T\times [e_{1}^{\varepsilon }(x_{i}),e_{2}^{\varepsilon }(x_{i}),e_{3}^{\varepsilon }(x_{i}),e_{4}^{\varepsilon }(x_{i}),e_{5}^{\varepsilon }(x_{i}),e_{6}^{\varepsilon }(x_{i}){]}^{T} \end{array}$$

where *T* denotes the conversion relationship between surface strain and section strain and *µ* denotes the Poisson ratio. *r* represents the outer radius of the section, as shown in Figure 1.

A section strain $e_{i}^{\varepsilon \;}\left(i=1,\;2,\ldots ,\;6\right)$ at any position on the neutral axis can be expressed as $\left(e_{1}^{\varepsilon },\;\;e_{2}^{\varepsilon },\;e_{3}^{\varepsilon },\;e_{4}^{\varepsilon },\;\;e_{5}^{\varepsilon },\;e_{6}^{\varepsilon }\right)$^T^ = R∙[$q_{1},q_{2},\;q_{3},\;q_{4},\;q_{5},\;q_{6}$]^T^. The matrix [$q_{1},q_{2},q_{3},q_{4},q_{5},q_{6}$]^T^ is used to represent the unknown parameters of the section strain. The specific form of the matrices R and [$q_{1},\;q_{2},\;q_{3},\;q_{4},\;q_{5},\;q_{6}$]^T^ are shown below.

(7) $$R=\left[\begin{array}{llllll} 1 & 0 & 0 & 0 & 0 & 0\\ 0 & y_{i} & 1 & 0 & 0 & 0\\ 0 & 0 & 0 & y_{i} & 1 & 0\\ 0 & \frac{D_{y}}{G_{z}} & 0 & 0 & 0 & 0\\ 0 & 0 & 0 & \frac{D_{z}}{G_{y}} & 0 & 0\\ 0 & 0 & 0 & 0 & 0 & 1 \end{array}\right]$$

(8) $$\begin{array}{ll} {}[q_{1},q_{2},q_{3},q_{4},q_{5},q_{6}{]}^{T} & =(T\times R^{u}{)}^{-1}\times \varepsilon^{a}(x_{i},\theta_{i},\beta_{i}{)}^{T}\\ & =(T\times \left[\begin{array}{llllll} 1 & 0 & 0 & 0 & 0 & 0\\ 0 & x_{i} & 1 & 0 & 0 & 0\\ 0 & 0 & 0 & x_{i} & 1 & 0\\ 0 & \frac{D_{y}}{G_{z}} & 0 & 0 & 0 & 0\\ 0 & 0 & 0 & \frac{D_{z}}{G_{y}} & 0 & 0\\ 0 & 0 & 0 & 0 & 0 & 1 \end{array}\right]{)}^{-1}\times \varepsilon^{a}(x_{i},\theta_{i},\beta_{i}{)}^{T} \end{array}$$

where *T* = [*T*_1_, *T*_2_, *T*_3_, *T*_4_, *T*_5_, *T*_6_]^T^. The difference between the *R* matrix and the *R^u^* matrix is that the variable *y_i_* in the *R* matrix is transformed into *x_i_*, and *x_i_* and *y_i_* denote a position at any point along the axis in the x-direction or y-direction.

By substituting the obtained sectional strain $e_{i}^{\varepsilon \;}\left(i=1,\;2,\ldots ,\;6\right)$ into Equation (4), the relationship between the nodal DOF of the structural and the surface strain can be established. This is shown in Equation (9).

(9) $$\begin{array}{l} u_{e}=(k^{e}{)}^{-1}f^{\mu }=T_{k}\times \varepsilon^{a}(x_{i})\\ T_{k}=\{\frac{l}{n}\times \sum_{i=1}^{6}[w_{p}M_{p}^{T}(x_{i})M_{p}(x_{i})]{\}}^{-1}\times \{\frac{l}{n}\times \sum_{i=1}^{6}\left[w_{p}M_{p}^{T}(x_{i})\right]R(T\times R^{u}{)}^{-1}]\} \end{array}$$

where the matrix *T_k_* is called the displacement–strain transformation matrix, which is determined by the position of the strain sensors after installation.

**Figure 2:**
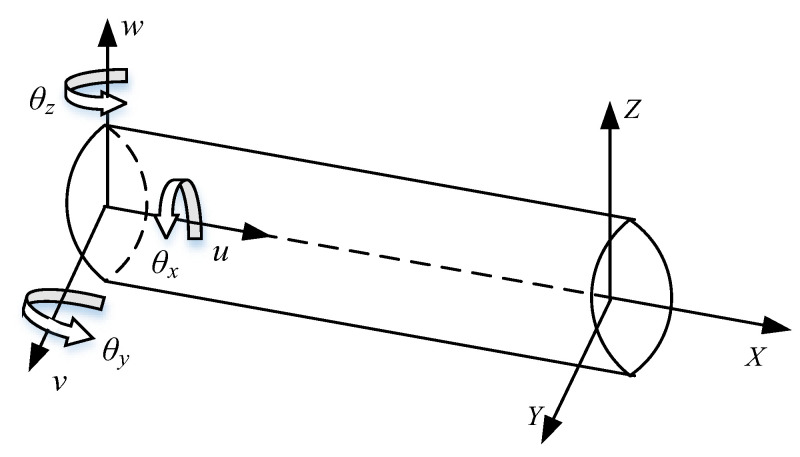
Geometric and kinematic variables of the structure.

As shown in Figure 2, taking the Timoshenko beam as an example, the deformation of this neutral axis is determined by the displacements *u*, *v* and *x* along each axis and the rotation angles *θ_x_*, *θ_y_* and *θ_z_* of each axis, the strain values are obtained from the strain sensors affixed to the surface of the structure, and the nodal DOFs are determined by Equations (4)–(9) and expressed as Equation (10):

(10) $$u=[u,v,w,\theta_{x},\theta_{y},\theta_{z}{]}^{T}$$

where *u*, *v*, *w*, *θ_x_*, *θ_y_* and *θ_z_* are also called the kinematic variables of the nodal DOFs *u*. When the nodal DOFs *u*(*x*) are determined, the shape function *H*(*x*) is obtained by interpolation of the nodal DOFs, and the displacement *d* of the final reconstruction is determined by the shape function and the nodal DOFs, as in Equation (11).

(11) $$d=\left\{\begin{array}{c} d_{x}\\ d_{y}\\ d_{z} \end{array}\right\}=\left[\begin{array}{llllll} 1 & 0 & 0 & 0 & z & -y\\ 0 & 1 & 1 & -z & 0 & 0\\ 0 & 0 & 1 & y & 0 & 0 \end{array}\right]\left[\begin{array}{l} u(x)\\ v(x)\\ w(x)\\ \theta_{x}(x)\\ \theta_{y}(x)\\ \theta_{z}(x) \end{array}\right]=H(x)u(x)$$

## 3. Coarse and Fine Two-Stage Calibration Method

### 3.1. Coarse Calibration

The selection of the proper sensor location is critical for accurate displacement and strain measurements. The sensor should be placed in the area that is most sensitive to the required measurement quantity in order to obtain the most accurate data. The fixation and alignment of the sensor during installation are also very important. If the sensor is not properly fixed or aligned, this may lead to unwanted errors or deviations. Therefore, ensuring good contact, stability and accuracy between the sensor and the object being measured is critical during the measurement process.

When the layout position of the strain sensor is determined, human installation factors will cause the sensor pasting position to offset, thus affecting the accuracy of the displacement–strain transformation matrix *T_k_* associated with the sensor position in Equation (9), resulting in an error Δ*T_k_* between the theoretical displacement–strain transformation matrix *T_k_* and the actual displacement–strain transformation matrix *T_k_^′^* after the sensor is installed, as shown in Equation (12).

(12) $${T_{k}}^{\prime }=T_{k}+\Delta T_{k}$$

To reduce the effect of sensor position offset on the reconstructed displacement accuracy, an error compensation method based on SOPSO is proposed for the displacement–strain transformation matrix.

The root mean square error (*RMSE*) is defined as:

(13) $$RMSE=\sqrt{\frac{1}{n}\sum_{m=1}^{n}\left(disp_{i}^{u}-disp_{i}^{v}(P^{\prime })\right)}$$

The relative root mean square error (*RRMSE*) is defined as:

(14) $$RRMSE=\frac{RMSE}{max(disp^{u})}\times 100\%$$

where *n* denotes the number of position sensors (check points). *P*′ denotes the actual installation positions of the 6 strain sensors (*i* = 1, 2, …, 6), which consists of the actual overall sensor layout position when the strain sensor installation position is offset. 

After the installation of the strain sensor, the strain value during the deformation of the structural body is measured, and the theoretical displacement value $disp_{i}^{v}$ is obtained based on the iFEM reconstruction. $disp_{i}^{u}$ denotes the actual measured displacement at check points.

The sensor position optimization model based on iFEM is shown in Equation (15):

(15) $$\begin{array}{l} Minmize\;Index(P^{\prime })=[RRMSE(P^{\prime })]\\ P^{\prime }=({P_{1}}^{\prime },{P_{2}}^{\prime },{P_{3}}^{\prime },{P_{4}}^{\prime },{P_{5}}^{\prime },{P_{6}}^{\prime }) \end{array}$$

where the exponent (*P*′) denotes the objective function of optimization. In this paper, the number of sensors required for the displacement field reconstructed is six [22].

Since the specific offset position cannot be obtained after installation, according to the actual situation, the optimization range can be set around the theoretical installation position of the sensor extracted in the simulation software ANSYS. The optimized sensor position parameter (x_i_*, θ_i_*, β_i_*) can be obtained based on SOPSO, and the modified displacement–strain matrix $T_{k}^{*}$ can be obtained by substituting the parameter (xi*, θi* and βi*) into Equations (6)–(9), improving the reconstruction accuracy of the displacement field.

Particle swarm optimization (PSO) is an evolutionary computational technique. All particles in the swarm adjust their velocity and position according to the current individual extreme value they find and the current global optimal solution shared by the whole swarm.

The basic idea of PSO is to search for optimal solutions to optimization problems through collaboration and information sharing among individuals in a population, as shown in Equations (16) and (17):

(16) $$V^{n+1}=V^{n}\times w+c_{1}\times rand\_f_{1}\times (X_{i}^{n}\_pbest-X_{i}^{n})+c_{2}\times rand\_f_{2}\times (X_{i}^{n}\_gbest-X_{i}^{n})$$

(17) $$X_{i}^{n+1}=X_{i}^{n}+V^{n+1}$$

where the inertia factor *w* is a value between 0 and 1. As the number of iterations increases, $X_{i}^{n}\;$denotes each position parameter of each particle, which in this paper means the position of the sensor. *c*_1_ and *c*_2_ denote acceleration factors; *rand_f*_1_ and *rand_f*_2_ are random values between 0 and 1; $X_{i}^{n}\_pbest$ is denoted as an individual optimum, indicating the optimal position of particle *X_i_* in the previous generation during the iterative process; and $X_{m}^{n}\_gbest\;$is denoted as a population optimum, indicating the best position explored by all individuals in the population.

**Figure 3:**
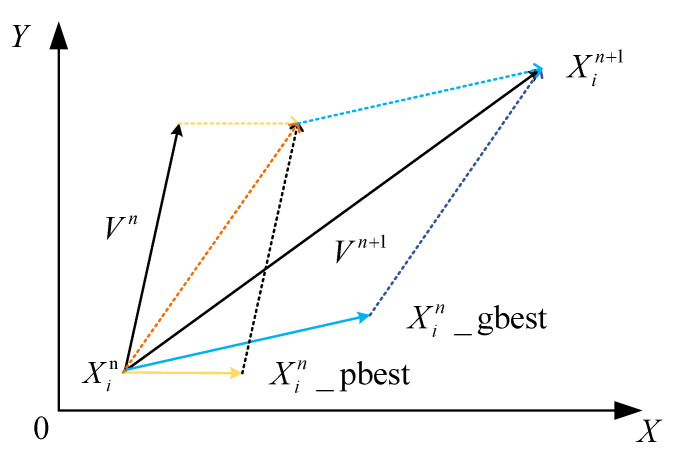
The transfer process of particle swarm optimization.

The fitness function *f*(*X_i_*) of PSO is obtained by solving the optimization model in Equation (15). The iterative process is shown in Equations (16) and (17), and the particle moves from *X_i_* to $X_{i}^{n}$ with the updated velocity *V^n^* as shown in Figure 3.

The steps for solving the optimization model (15) based on the single-objective particle swarm algorithm are shown below.

Step 1: The initial position *X*_0_ and initial velocity *V*_0_ of the particle swarm algorithm are initialized, setting them within the installation error of the sensor. The number of the population size is set to *N* = 80, and the maximum number of iterations is *n_max_* = 100.

Step 2: In each generation of evolution, the value of the fitness function *f*($X_{i}^{1}\_pbest$) is calculated for each particle, the values are compared to see whether the current fitness value of each particle is better than the historical local optimum, i.e., *f*($X_{i}^{1}$) > *f*($X_{i}^{0}\_pbest$), the current particle fitness value *f*($X_{i}^{1}$) is taken as the local optimum of the particle, and then its current position is taken as the optimal position of the particle. The current fitness value of each particle is compared with the fitness value *f*($X_{m}^{n}\_gbest$) corresponding to the global best position $X_{m}^{n}\_gbest$; if the current fitness value is higher, the global best position will be updated with the current particle position.

Step 3: the algorithm iterates to the maximum number of iterations, *n = n_max_*, and the algorithm terminates.

In summary, we call this method of displacement error calibration coarse calibration. On the basis of the coarse calibration method, fine calibration is performed in Section 3.2 to further improve the reconstruction accuracy of the deformation field.

### 3.2. Fine Calibration

#### 3.2.1. The Nodal Degrees of Freedom Error Correction

For the errors that still exist between the displacements reconstructed based on the iFEM and the actual displacements after the coarse calibration, the displacement nodal degrees of freedom error correction model is further proposed. As shown in Equation (18), the theoretical displacement of the iFEM reconstruction is denoted as $d_{i}^{t}$, and the actual displacement is denoted as $d_{i}^{a}$*:*

(18) $$d_{i}^{a}=d_{i}^{t}+\Delta d_{i}$$

where *i* = 1, …, n denotes the ordinal number of observation points and Δ*d_i_* denotes the reconstruction error. Displacement at any position in the structure can be obtained from the shape function *H*(*x*) and the discrete nodal during the reconfiguration displacement. As a result, the whole displacement field can be calibrated by correcting the reconstruction error of the nodal DOFs.

The relationship between displacement error and nodal DOFs error can be expressed as Equation (19).

(19) $$\Delta d_{i}=\left[\begin{array}{c} \Delta d_{i}^{x}\\ \Delta d_{i}^{y}\\ \Delta d_{i}^{z} \end{array}\right]=G\times N_{i}\times \Delta u$$

where the number of nodal DOFs is c. The displacement field error Δ*d_i_* of the inverse finite element reconstruction is derived by Equation (19) to the nodal DOFs error Δ*u_j_*(*j* = 1, …, c), and then the displacement nodal DOFs model can be expressed as Equation (20)

(20) $$\Delta d^{z}=H\cdot \Delta u+\beta$$

where Δ*d^z^* = [Δ$d_{1}^{z}$, Δ$d_{2}^{z}$, *…*, Δ$d_{n}^{z}$]^T^, *H* = [*H*_1_*.H*_2_, *…*, *H_n_*], *β* is denoted as the residuals after displacement error correction and obeys Gaussian distribution, i.e., $\beta \sim N(0,\sigma^{2})$, and $H_{i}=[H_{i1},H_{i2},\cdots ,H_{ic}]$ is the row vector of G*N_i_ for Δd^z^. It follows that Δd^x^ and Δ*d^y^* can also be expressed analogously by Equation (20).

The matrix *H* in Equation (20) is the ill-conditioned matrix. The actual measured strain value of the sensor receives interference from random noise, which makes the estimated value Δ*u* have an error with the actual value, and the error is reduced by the Bayesian regularization algorithm using prior information. From Bayesian theory, *p*(Δ*u*, *σ*^2^*|*Δ*d^x^*)is used to represent the joint posterior distribution of the unknown parameters, as defined as shown in Equation (21):

(21) $$p(\Delta u,\sigma^{2}|\Delta d^{x})\propto p(\Delta d^{x}|\Delta u,\sigma^{2})\cdot p(\Delta u,\sigma^{2})$$

where *p*(Δ*u*,*σ*^2^) denotes the joint prior distribution, rewriting Equation (21) into multiplicative form, which assumes that the residuals are independently Gaussian distributed, i.e., $\beta =\left[\Delta d^{x}-H\cdot \Delta u\right]\tilde{N}\left(0,\sigma^{2}\right)$, and that the non-informative prior of *σ*^2^ is taken into account, i.e., $p\left(\sigma^{2}\right)\propto 1/\sigma^{2}$. Assuming that the prior distribution of Δ*u* is Gaussian, i.e., $p\left(\Delta u\right)=N\left(0,\tau^{2}\right)$, the posterior distribution in logarithmic form $\ln p=(\Delta u,\sigma^{2}|\Delta d^{x})$ is represented:

(22) $$lnp(\Delta u,\sigma^{2}|\Delta d^{z})\propto -\sum_{i=1}^{n}[\Delta d_{i}^{z}-H_{i}\cdot \Delta u{]}^{2}-\lambda \sum_{j=1}^{c}{\left\|\Delta u_{j}\right\|}^{2}$$

where $\lambda =\frac{\sigma^{2}}{\tau^{2}}$, and the conditional posterior distribution of Δ*u* in Equation (23) is calculated using the conjugate distribution method as *N*(Δ*u_j_*_,_$\Delta s_{j}^{2}$).

(23) $$\begin{array}{l} \Delta u_{j}=(\sum_{i=1}^{n}x_{ij}^{2}+\lambda {)}^{-1}\sum_{i=1}^{n}x_{ij}(\Delta d_{i}^{z}-\sum_{j=1,j\neq j}^{c}x_{ij}\Delta u_{j})\\ s_{j}^{2}=(\sum_{i=1}^{n}x_{ij}^{2}{)}^{-1}\sigma^{2}+\tau^{2} \end{array}$$

For σ^2^, the conditional posterior distribution is the inverse chi-square distribution, expressed as:

(24) $$\sigma^{2}\text{\textasciitilde{}}\frac{1}{\chi^{2}}{\sum_{i=1}^{n}\left(\Delta d_{i}^{z}-\sum_{j=1}^{c}x_{ij}\Delta u_{j}\right)}^{2}$$

**Figure 4:**
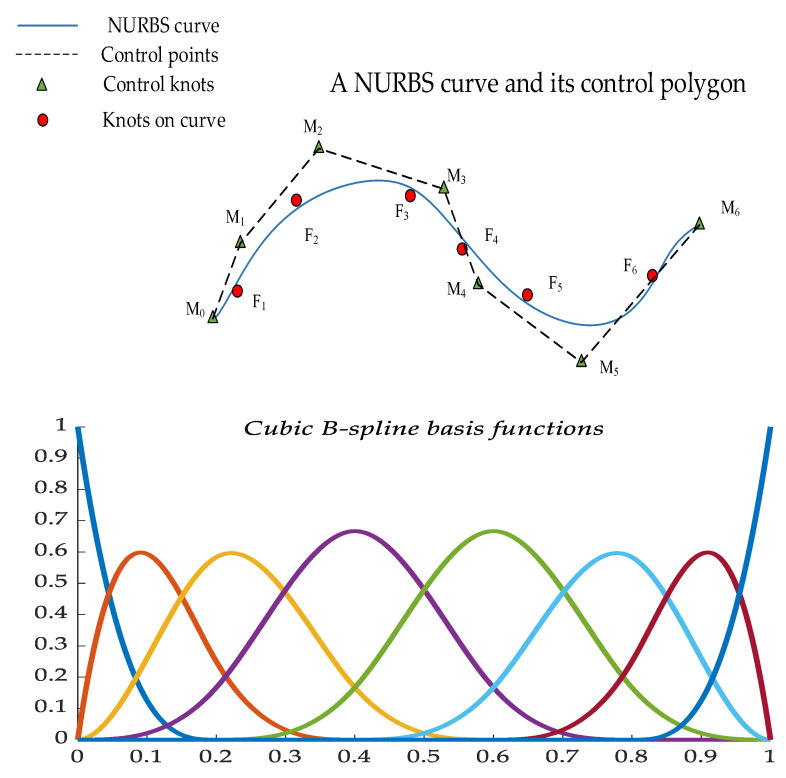
NURBS approximation diagram.

Therefore, Markov chain Monte Carlo (MCMC) samples are produced from the posterior *p*(Δ*u*,*σ*^2^*|*Δ*d^x^*) via a Gibbs sampler for calibration amounts Δ*u*, and the steps are as follows: (1) initialize Δ*u* and *σ*^2^; (2) perform Gibbs sampling on Δ*u* in Equation (23); and (3) select the inverse chi-square distribution from Equation (24), *σ*^2^. The specific process details are shown in Figure 4.

The calibration values of each kinematic variable in the nodal DOFs error are derived from the Bayesian regularization algorithm and are combined with the actual measured strain values to form the sample data, defined in Equation (25).

(25) $$Q_{j}=\{{\left(\varepsilon_{1},\varepsilon_{2},\ldots ,\varepsilon_{L}\right)}_{j},{\left(\Delta u_{1},\Delta u_{2},\ldots ,\Delta u_{m}\right)}_{j}\}$$

where *Q_j_* denotes the *j*th (*j* = 1, 2, …, h) sample data under working conditions and $\varepsilon_{l}$ denotes the *l*th (*l* = 1, 2, …, L) strain value measured by the sensor. ∆*u_i_* (*i* = 1, 2, …, m) denotes the error calibration value of the kinematic variable in the nodal DOFs error.

#### 3.2.2. Training Data Filtering and Sample Size Expansion

The error correction method has limitations: the nodal DOFs errors can only be calibrated under specific working conditions. Therefore, in order to obtain the error calibration between strain values and nodal DOFs under arbitrary working conditions, the SCFN is used for calibration. The selection of appropriate training data affects the training effect of SCFN, and this paper employed residual analysis to screen the sample data. In addition, the training effect of the SCFN is also related to the number of training samples, so the number of training samples should be expanded before entering the training network.

The residual is the difference between the actual observed value and the fitted value of the sample. The reliability and reasonableness of the data are analyzed using the residual, defined as follows.

(26) $$e=y-\hat{y}$$

where *y* represents the actual observed value, $\hat{y}$ represents the fitted value, and *e* represents the residual.

In this paper, considering that high-dimensional data can cause excessive computation, the screening of sample data requires residual analysis for each kinematic variable in the calibration values of DOFs to ensure the reasonableness of screening training data.

The new sample point is obtained by decomposing the original sample data *Q_j_* in Equation (25):

(27) $$\left\{ \begin{array}{l} F_{1}=\{(\varepsilon_{1},\varepsilon_{2},\cdots ,\varepsilon_{l}{)}_{1},\Delta u_{1}\}\\ F_{2}=\{(\varepsilon_{1},\varepsilon_{2},\cdots ,\varepsilon_{l}{)}_{2},\Delta u_{2}\}\\ \;\;\;\;\;\;\;\;\;\;\;\;\vdots \\ F_{m}=\{(\varepsilon_{1},\varepsilon_{2},\cdots ,\varepsilon_{L}{)}_{m},\Delta u_{m}\} \end{array}\right.$$

Each sample point is decomposed into *F_i_* (*i* = 1, 2, …, m) of the form, where each vector *F_i_* consists of *L* measured strain values $\varepsilon_{L}$ and 1 kinematic variable of nodal DOFs error, and the kinematic variables are denoted as Δ*u_i_* (*i* = 1, 2, …, m).

The sample fit values are obtained before the sample data are screened based on the magnitude of the residuals. In this paper, the nonuniform rational B-spline (NURBS) curve fitting method is used to fit the data *F_i_*, as shown in Figure 4. 

The NURBS curve of order *c* can be expressed as a segmented rational polynomial vector function, and the mathematical definition is as follows.

(28) $$p(t)=\frac{\sum_{i=0}^{h}k_{i}M_{i}E_{i,c}(t)}{\sum_{i=0}^{h}k_{i}E_{i,c}(t)}$$

where *k_i_* (*i* = 0, 1, …, *h*) denotes the weight factors, associated with the control vertex *M_i_* (*i* = 0, 1, …, *h*); due to the lack of expert experience, the weighting constants *k_i_* = 1 (*i* = 0, 1, …, *h*). *E_i_*_,*c*_(*t*) denotes the *c*-th order B-spline basis function, which is a non-decreasing sequence of parameters. *T =* [*t*_0_, *t*_1_, …, *t_h+c+_*_1_] is the nodal vector and determined by the *c*-th-order segmented polynomial.

The basis function can be recursively expressed by:

(29) $$\begin{array}{l} E_{i,0}(t)=\{\begin{array}{c} 1,t_{i}\leq t\leq t_{i+1}\\ 0,otherwise \end{array}\\ E_{i,c}(t)=\frac{t-t_{i}}{t_{i+c}-t_{i}}E_{i,c-1}(t)+\frac{t_{i+c+1}-t}{t_{i+c+1}-t_{i+1}}E_{i+1,c-1}(t) \end{array}$$

The parameterization of the data points reflects the nature of the curve constructed with the data points. Based on the nodal vector of basis functions and control points to construct the NURBS fitted curves, a centripetal parameterization method is applied to the sample data as shown in Equation (30).

(30) $$\left\{ \begin{array}{l} {\bar{t}}_{0}=0\\ {\bar{t}}_{i}={\bar{t}}_{i-1}+\frac{|F_{i}-F_{i-1}{|}^{\frac{1}{2}}}{\sum_{i=1}^{h}|F_{i}-F_{i-1}{|}^{\frac{1}{2}}}\\ {\bar{t}}_{h}=1 \end{array}\right.(i=1,2,\cdots ,h-1)$$

As required by the definition domain of the non-closed curve, multiple knots with repetition degree *c* + 1 are taken at the two endpoints of the definition domain. According to the distribution of the sample and the method of centripetal parameterization, the nonuniform vector method is used for the construction, shown in Equation (31).

(31) $$\left\{ \begin{array}{l} t_{0}=t_{1}=\cdots =t_{c}=0\\ t_{j+c}=\frac{1}{c}\sum_{i=j}^{j+c-1}{\bar{t}}_{i}\;\;\;\;\;\;\;\;\;\;(j=1,2,\cdots ,h-c)\\ t_{h+1}=t_{h+2}=\cdots =t_{h+c+1}=1 \end{array}\right.$$

The *c*-th-order NURBS curve is constructed by *m* + 1 sampling points *F_i_* (*i* = 0, 1, …, *m*). The approximation function is obtained by using the least squares method to approximate the parameter sequence $\bar{t_{i}}\;$(*i* = 0, 1, …, *m*), shown in Equation (32).

(32) $$\delta (p,\bar{t})={\sum_{i=1}^{m}\left\|F_{i}-p(\bar{t_{i}})\right\|}^{2}$$

where the control points are set to *A*_0_ = *F*_0_, *A_m_* = *F_h_*, and then *S_i_* = *F_i_ − F*_0_*E*_0,*c*_(${\bar{t}}_{i}$) *− F_h_E_p_*_,*c*_(${\bar{t}}_{i}$). The partial derivatives of the control points in Equation (32) are equal to 0.

The matrix form is expressed as:

(33) $$M(E^{T}E)=S$$

where the *E* matrix is a matrix with *h* − 1 rows and *m* − 1 columns and *E^T^* is the transpose matrix of *E*. The *E*, *S* and *M* matrices are shown in Equation (34).

(34) $$\begin{array}{c} E=\left[\begin{array}{cccc} E_{1,c}(\bar{t_{1}}) & E_{2,c}(\bar{t_{1}}) & \cdots & E_{h-1,c}(\bar{t_{1}})\\ E_{1,c}(\bar{t_{2}}) & E_{2,c}(\bar{t_{2}}) & \cdots & E_{h-1,c}(\bar{t_{2}})\\ \vdots & \vdots & \ddots & \vdots \\ E_{1,c}(\bar{t_{m-1}}) & E_{2,c}(\bar{t_{m-1}}) & \cdots & E_{h-1,c}(\bar{t_{m-1}}) \end{array}\right]\\ S={\left[\begin{array}{cccc} E_{1,c}(\bar{t_{1}}) & E_{2,c}(\bar{t_{1}}) & \cdots & E_{h-1,c}(\bar{t_{1}})\\ E_{1,c}(\bar{t_{2}}) & E_{2,c}(\bar{t_{2}}) & \cdots & E_{h-1,c}(\bar{t_{2}})\\ \vdots & \vdots & \ddots & \vdots \\ E_{1,c}(\bar{t_{m-1}}) & E_{2,c}(\bar{t_{m-1}}) & \cdots & E_{h-1,c}(\bar{t_{m-1}}) \end{array}\right]}^{T}\cdot \left[\begin{array}{c} s_{1}\\ s_{2}\\ \vdots \\ s_{m-1} \end{array}\right]\\ \;\;\;\;\;\;\;\;\;\;\;\;\;\;\;\;\;M=\left[\begin{array}{c} M_{1}\\ M_{2}\\ \vdots \\ M_{h-1} \end{array}\right] \end{array}$$

The specific steps to obtain the sample fit values using NURBS curve fitting are as follows:

(1) The data are parameterized by Equation (29) to obtain ${\bar{t}}_{i}$ (*i* = 1,2,…,*m*) and the basis function *E_i_*_,_ *_c_*(*t*).
(2) After obtaining ${\bar{t}}_{i}$, the nodal vector *T* is obtained by Equation (30).
(3) The control point *M_i_* (*i* = 1,2,…,*h*) is obtained according to Equation (33).
(4) The constructed NURBS fitting curve is then derived from Equation (28).
(5) When the NURBS fitted curve is obtained, the values of the sample data parameters are substituted into the curve equation of Equation (28), and then the values on the fitted curve corresponding to the original sample data can be obtained.

Similarly, the fitted data $\hat{F}$*_i_* (*i* = 1, 2, …, *m*) for the rest of the samples under a working condition can be obtained as shown below.

(35) $$\left\{ \begin{array}{l} {\hat{F}}_{1}=\{({\hat{\varepsilon }}_{1},{\hat{\varepsilon }}_{2},\cdots ,{\hat{\varepsilon }}_{L}{)}_{1},\Delta {\hat{u}}_{1}\}\\ {\hat{F}}_{2}=\{({\hat{\varepsilon }}_{1},{\hat{\varepsilon }}_{2},\cdots ,{\hat{\varepsilon }}_{L}{)}_{2},\Delta {\hat{u}}_{2}\}\\ \;\;\;\;\;\;\;\;\;\;\;\;\vdots \\ {\hat{F}}_{m}=\{({\hat{\varepsilon }}_{1},{\hat{\varepsilon }}_{2},\cdots ,{\hat{\varepsilon }}_{L}{)}_{m},\Delta {\hat{u}}_{m}\} \end{array}\right.$$

When screening training data using residual analysis, the error in the kinematic variables Δ*u_i_* (*i* = 1, 2, …, *m*) varies considerably in order of magnitude due to the actual situation, which affects the residual analysis. The transformation matrix *H* in Equation (2) is related to the observed coordinates of the position sensor (check point) and other factors, and the order of magnitude of each column of the transformation matrix *H* varies greatly. According to Equation (19), Δ*d_i_* for the same working conditions is generally under the same order of magnitude, so it leads to kinematic variable errors Δ*u_i_* (*i* = 1, 2, …, *m*) obtained from the solution of different orders of magnitude.

According to the analysis described above, the main factor affecting the magnitude of the kinematic variable error is the column vector of transformation matrix *H*. Therefore, the weighting constants are calculated as shown in Equation (36).

(36) $$a_{i}=\frac{1}{v}\sum_{k=1}^{v}H_{ki}$$

where *a_i_* (*i* = 1, 2, …, *m*) denotes the weighting constants corresponding to the *m*-th kinematic variable error and *H_ki_* denotes the *k*-th row and *i*-th column of the transformation matrix.

When the position of the position sensor is fixed, the matrix *H* is constant under any working condition, so the weighting constants corresponding to the kinematic variable error are also unchanged. At this time, the integrated residual value *e^t^* of the sample under one working condition can be obtained from the Euclidean distance, which is calculated as shown in Equation (37).

(37) $$e^{t}=\sqrt{{\sum_{i=1}^{m}\left(a_{i}\cdot (\Delta u_{i}-\Delta {\hat{u}}_{i})\right)}^{2}}$$

In summary, the integrated residuals of the samples $e_{j}^{t}$ (*j* = 1, 2, …., *h*) under the remaining working conditions can be obtained.

The screened training data consist of three parts: boundary samples, representative samples and normal samples after equidistant sampling. The boundary samples are the sample points where the structure is in the minimum and maximum load states in the experiment. After extensive experiments and summaries, the conclusion can be drawn that the rest of the sample points in the normal sample points are values with a small range of residual fluctuations, and the residual values of representative sample points are more than twice the fluctuation range of the residual values of normal sample points.

The specific steps of the screening training data method are as follows: (1) The NURBS curve fitting method is adopted to obtain the corresponding fitting data of sample points. (2) The corresponding fitting data are subtracted from the sample data to obtain the residual values of each sample point under different working conditions. (3) The boundary samples, representative samples and normal samples obtained by equidistant sampling are selected as the training samples for constructing the self-constructed fuzzy neural network. The training sample set *F^sc^* screened from all samples *Q_j_* is denoted as:

(38) $$F^{sc}=\{F_{1}^{sc},F_{2}^{sc},\cdots ,F_{o}^{sc}\}\;o\leq j$$

The number of screened training samples $F_{o}^{sc}$ is expanded through the NURBS curve fitting method. After obtaining the fitting curve, a specific step is taken to extend the sample quantity by substituting values of ${\bar{t}}_{i}$ ranging from 0 to 1 into Equation (28). Then, a self-constructed fuzzy network is trained based on the extended samples.

#### 3.2.3. Self-Constructed Fuzzy Network Calibration

Due to the nonlinear and coupling relationship between the strain values in the sample data and the nodal DOFs calibration values, a mathematical model is difficult to build. However, SCFN can effectively solve this nonlinear problem. After screening all samples for suitable training samples and extending them, the SCFN is trained based on the extended samples. A strain nodal DOFs calibration model is established. The SCFN enhances the generalization ability of the strain nodal DOFs calibration value and improves the accuracy of the reconstructed displacement by calibrating the nodal DOFs. SCFN is divided into two steps [32]: (1) adding the affiliation function and generating the corresponding fuzzy rules and (2) adaptively adjusting the results of the fuzzy rules.

In this paper, the triangular affiliation function has a simple structure, which is convenient and efficient for calculating the membership degree. Thus, SCFN chooses the triangular membership function and the 0th-order T-S fuzzy model, which is represented by the fuzzy rule set as follows.

(39) $$S^{n}:\;if\;\varepsilon_{1}\;is\;R_{1}^{n}\;and\;\varepsilon_{2}\;is\;R_{2}^{n}\;\cdots and\;\varepsilon_{K}\;is\;R_{K}^{n}\;then\;{\hat{p}}^{n}\;=w^{n}$$

where *S^n^* (*n* = 1, 2, …, *N*) denotes the nth fuzzy rule, $\varepsilon_{k}$ (*k* = 1, 2, …, *K*) is denoted as the input strain value, and $R_{n}^{k}$ denotes the membership degree corresponding to $\varepsilon_{k}$ in the nth rule. ${\hat{p}}^{n}$ denotes the output of the nth fuzzy rule, and $w^{n}$ is the value corresponding to its fuzzy rule output.

The system output of SCFN $\hat{p}$ represents the nodal DOFs error, and when a certain input activates *m* rules (*n* ≥ *m*), the system output is derived from the ${\hat{p}}^{n}$ of these *m* outputs by weighted averaging, as shown in Equation (33).

(40) $$\hat{Y}=\frac{\sum_{n=1}^{m}q_{n}{\hat{p}}^{n}}{\sum_{n=1}^{m}q_{n}}=\frac{q_{1}{\hat{p}}^{1}+q_{2}{\hat{p}}^{2}+\cdots +q_{m}{\hat{p}}^{m}}{q_{1}+q_{2}+\cdots +q_{m}}$$

The $q_{n}$ in Equation (40) denotes the weighting constants of the rule, and the weighting constants are calculated by taking the smallest method, as shown in Equation (41).

(41) $$q_{n}=R_{1}^{n}\wedge R_{2}^{n}\wedge L\wedge R_{K}^{n}=\overset{K}{\underset{k=1}{\wedge }}R_{k}^{n}(\varepsilon_{k})$$

The SCFN is initialized with one rule, and the membership functions and rule number are added or removed based on the error and completeness criteria. When the number of training samples is *k* + 1, the error standard of SCFN is represented by root mean square error *η_t_*, and the formula is shown below.

(42) $$\eta_{t}=\sqrt{\frac{{\sum_{c=1}^{k}\left(\hat{U}(c)-U(c)\right)}^{2}}{k}}$$

where denotes the actual output, *U*(*c*) denotes the desired output, *η_a_* is denoted as a predetermined error threshold, and if *η_t_* > *η_a_*, it means that the system error does not meet the requirements and the membership function needs to be increased.

In SCFN, each input value at least corresponds to one membership function. If its membership degree $R_{k}^{n}$ is greater than the completeness threshold α, the completeness of the membership function meets the requirements; if $R_{k}^{n}$ is less than α, then membership functions and fuzzy rules need to be added. The completeness threshold is generally set to 0.5.

After adding the membership function and fuzzy rule, the fuzzy rule also needs to be adaptively adjusted to make it closer to the output value. The specific expression for adjusting the *n*th rule of the fuzzy controller at the *i*-th moment is as follows.

(43) $$a^{n}(i)=a^{n}(i-1)+V\cdot \varphi^{n}(i-1)\cdot (U(k-1)-\hat{U}(k))$$

where *a^n^*(*i*) denotes the rule adjustment result at moment *i*, *V* is used to adjust the adaptive speed during rule adjustment, *φ^n^*(*i −* 1) denotes the weighting constants of the nth rule at moment *i −* 1, *P*(*i −* 1) denotes the desired output of the nth rule at moment *i −* 1, and (*i*) is the current system output.

Each rule is added and adaptively adjusted according to the standard correspondence and then used to train the SCFN. when *η_t_* ≤ *η_a_*, the training is stopped to jump out of the iteration and generate the fuzzy network, and the trained fuzzy rules are saved to obtain the calibrated nodal freedom results.

**Figure 5:**
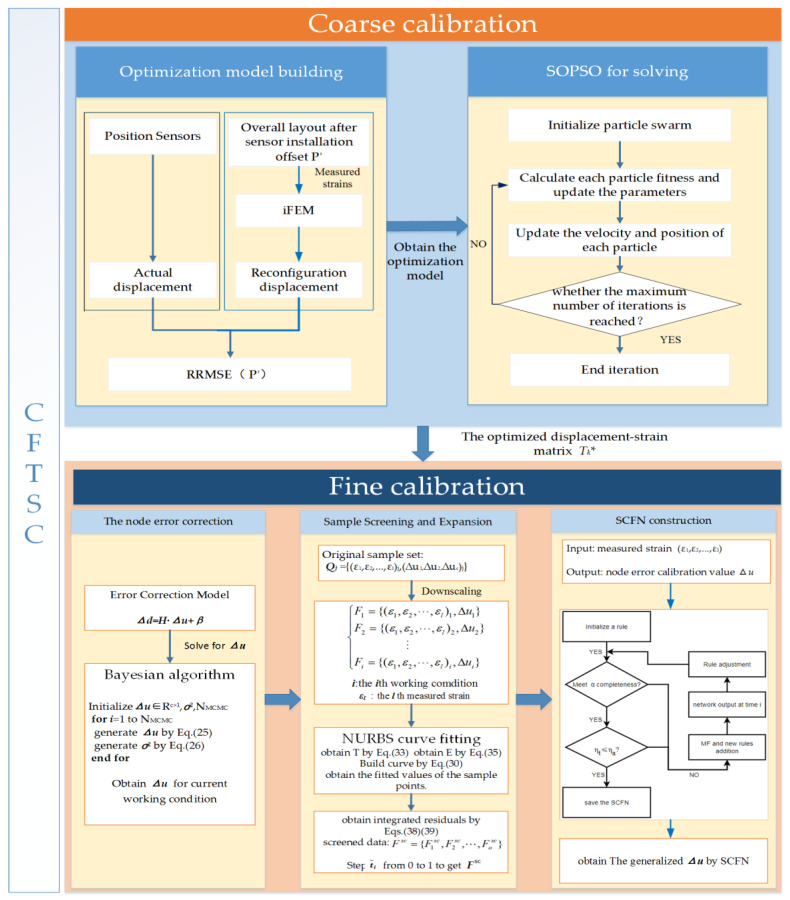
Flow framework of CFTSC method.

Summarizing the above, the method flow framework for the CFTSC method is shown in Figure 5.

## 4. Experimental Research

**Figure 6:**
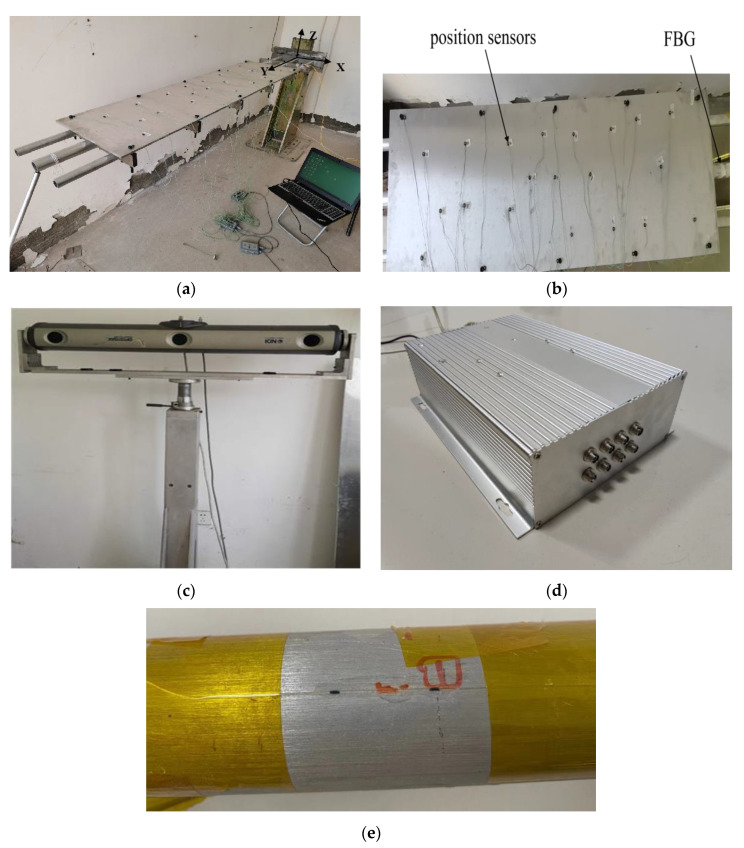
The whole experimental framework: (**a**) Experimental subject; (**b**) Position sensors and FBG; (**c**) Displacement-measuring instrument NDI; (**d**) Fiber Bragg grating demodulation system; (**e**) FBG sensor.

In order to verify the effectiveness of the CFTSC method proposed in this paper, the wing-integrated conformal antenna structure was considered as the experimental object. Experimental subjects were a plate and beam integrated structure, and the structure body consisted of three beams and an aluminum plate. The beams and plate were connected by six steel bars, and three beams were connected with fixed ends. Each beam was made of aluminum alloys and had a length of 2000 mm. The beam located in the middle of the three beams had an outer radius of 40 mm and an inner radius of 32 mm. The remaining two beams located on the sides had an outer radius of 30 mm and an inner radius of 24 mm. The specification of the plate in the structure was an aluminum plate with a length of 1120 mm, a width of 400 mm, and a thickness of 8 mm, placed and attached above the three beams with the plate 500 mm from the fixed end, as shown in Figure 6a.

With the deformation of the structural body, the Fiber Bragg grating (FBG) sensor installed on the beam (Figure 6e) transmitted the wavelength change information to the demodulator in Figure 6d, which was processed and input to the host computer as strain information, then the host computer calculated the strain value during the displacement of the structure, and, finally, the displacement was calculated in real time. Meanwhile, the displacement of the experimental subject was measured by Northern Digital Incorporation (NDI) shown in Figure 6c and taken as the actual displacement. In Figure 6b, the information on the position sensors (marked points) installed on the surface of the aluminum plate was collected by the 3D optical measuring device NDI, which has a measurement accuracy of approximately 0.1 mm and a resolution of 0.01 mm. The position sensors have a maximum emission frequency of 4600 Hz and a maximum acquisition frequency of 4600/(n + 1.3) Hz (n is the number of position sensors). The position sensors can obtain the deformation of the structure dynamically in real time to obtain the three-dimensional coordinates in the X-, Y- and Z-directions. As shown in Figure 6a, the coordinate system in X-, Y-, and Z-directions was established at the fixed end of the structure.

**Figure 7:**
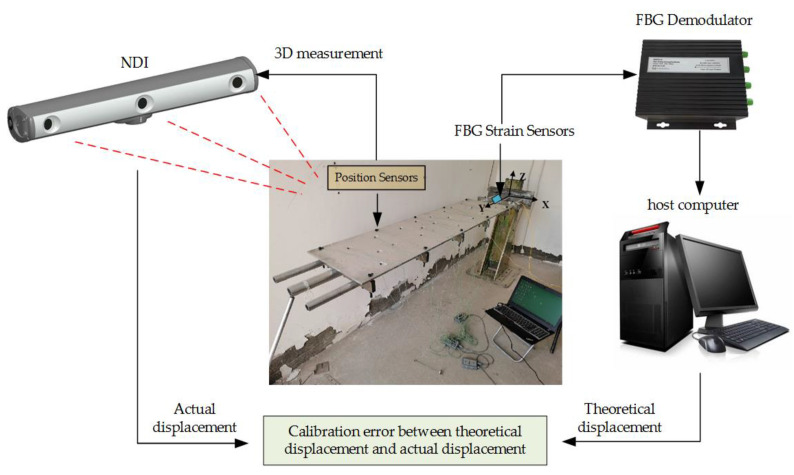
Functional diagram of each component of the experiment.

The specific information of the sensor installation was shown in Table 1. *x_p_* indicates the relative position of the sensor on the beam, and (*θ*, *β*), respectively, indicate the circumferential angle on the circular section and the angle with the X-axis when the sensor was installed. The functions and interconnections between the various pieces of hardware in this experiment are shown in Figure 7.

**Figure 8:**
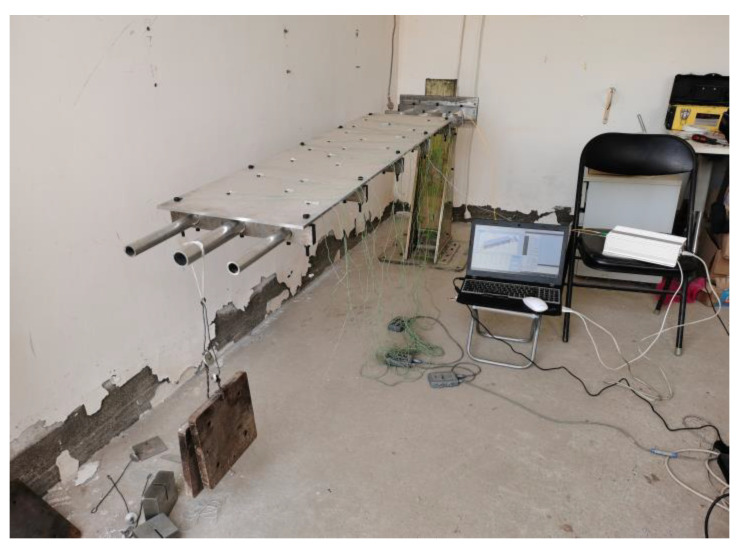
Model loading.

From Table 2, the position information of the 10 position sensors installed after establishing the coordinate system can be obtained. In this experiment, the strain values collected by the FBG sensor through the demodulator system and the position information collected by the position sensor of NDI are performed simultaneously, and Figure 8 shows the overall system of the experiment.

Relative to the fixed end, the load was added to the main beam at the other end of the structure (free end), as shown in Figure 8, and the magnitude of the static load under all working conditions is shown in Table 3. The CFTSC method calculates the displacement under various working conditions based on the strain values collected according to the concentrated load forces listed in Table 3.

The experiments of coarse calibration were first performed to verify the calibration effect. The information was obtained by the FBG demodulator system, and the theoretical displacement values of the structure at the 10 position sensors were reconstructed based on the iFEM. The actual deformation was measured by NDI, and the index was obtained from Equation (15).

In the simulation, high-fidelity finite elements of discontinuous beams were modeled with ANSYS to verify the effectiveness of the proposed method. The length of the girder beam *L*_1_ = 2000 mm, outer diameter R_1_ = 40 mm and inner diameter r_1_ = 32 mm; the length of the auxiliary girder *L*_2_ = 2000 mm, outer diameter R_2_ = 30 mm and inner diameter r_2_ = 24 mm. The distance of the center of the circle *d* = 130 mm, and the dimensions of the ribs are *l* = 400 mm, *b* = 50 mm and *h* = 20 mm. The specifications of the plate were a length of 1120 mm, width of 400 mm and thickness of the aluminum plate of 8 mm, which was divided into 6000 elements. Among the model, the ribbed plates are of steel construction, and the beams are aluminum products. It was modeled with 56,350 elements, including beams and solids. Table 4 presents the material properties of the modeled structure.

**Figure 9:**
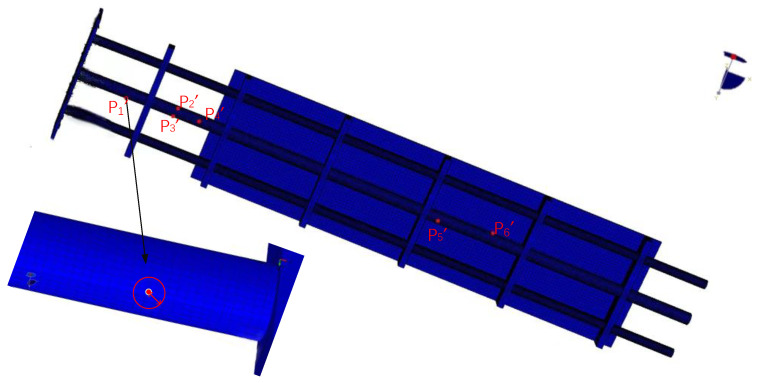
Example of strain sensor optimization range.

The strain sensors are manually installed according to the layout determined in the simulation software ANSYS. Based on engineering practice, the error of the position offset between the actual installation position and the theoretical installation position in ANSYS was within 1 cm. Therefore, the particle swarm optimization range of each strain sensor position information was set to be within 1 cm around the theoretical position in ANSYS and within ±30° of the angle. Taking strain sensor P_1′_ as an example, as shown in Figure 9, the optimization range for the remaining sensors was the same.

A total of 10 positions of position sensors were selected as check points to calculate RRMSE. The corrected sensor position parameters (xi*, θi* and βi*) can be obtained based on SOPSO. The maximum number of iterations n_max_ = 100, population size *N* = 80, inertia weight parameter *w* = 0.7, self-learning factor c_1_ = 1.49 and population learning factor c_2_ = 1.49. The corrected sensor position parameters (xi*, θi* and βi*) can be substituted into Equations (6)–(9) to obtain the corrected displacement strain matrix $T_{k}^{*}$, thus improving the reconstruction accuracy of the displacement field.

In order to test the effect of the coarse calibration method on the reconfiguration accuracy effect, indicators for estimating the calibration accuracy are proposed, which are defined as:

(44) $$RMSE=\sqrt{\frac{{\sum_{i=1}^{N}\left(disp^{n}\left(i\right)-disp^{c}\left(i\right)\right)}^{2}}{N}}$$

where the superscript ‘*n*’ indicates the actual displacement measured by NDI, ‘*c*’ indicates the calibrated displacement by the coarse calibration method, and ‘*m*’ indicates the reconstructed displacement by the iFEM. N indicates the number of position sensors.

**Figure 10:**
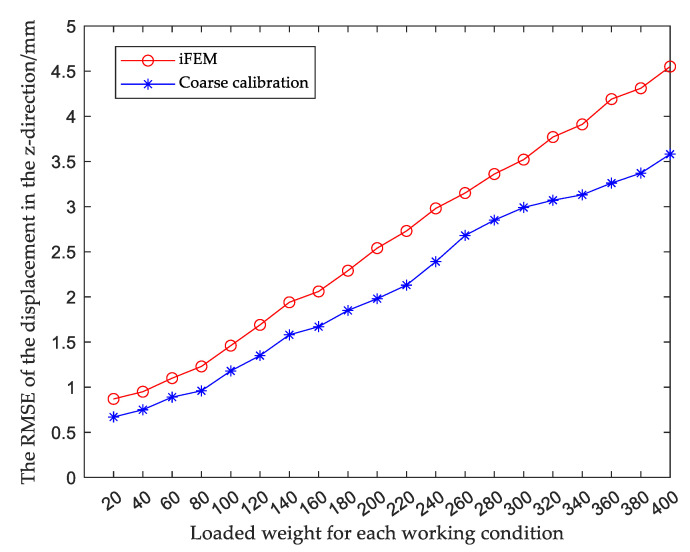
Comparison of displacement accuracy between coarse calibration and iFEM.

When the structure was in a certain working condition, the relative root mean square error (RMSE) in the z-direction of the main deformation direction after coarse calibration was obtained from Equation (44); similarly, the RMSE in the rest of the working conditions could be obtained and compared with the RMSE obtained by reconstructing the displacement using the iFEM, as shown in Figure 10, and the specific data are shown in Table 5.

The results show that compared with traditional iFEM, the calibration accuracy of the observed values for each working condition was improved by using the coarse calibration method. Next, fine calibration was performed on the basis of the coarse calibration to further improve the displacement reconstruction accuracy.

On the basis of the modified $T_{k}^{*}$ matrix obtained by coarse calibration, the displacement field was reconstructed by the iFEM. The reconstructed displacements are compared with the actual displacements obtained from the 3D optical measuring instrument NDI, the reconstructed displacement error Δ*d_i_* is obtained from Equation (18), and then the nodal DOFs error Δ*u* is obtained from Equation (20).

**Figure 11:**
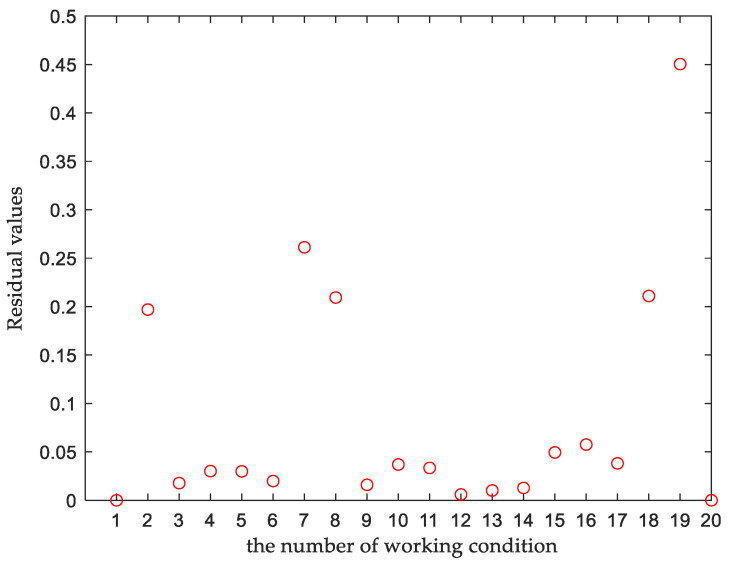
Residual plot for each working condition.

After the original sample data were decomposed, the fitted values of each kinematic variable were obtained by NURBS fitting, the corresponding weighting constants of each kinematic variable were calculated by Equation (36), the integrated residuals of the kinematic variable under each working condition were obtained by Equation (37), the residual plots for each working condition are shown in Figure 11. The integrated residuals of these 20 sets of working conditions were analyzed, and the residual values of most samples fluctuated in the range of 0–0.1, which was set as normal samples; samples with residual values 2 times those of normal samples or greater were set as representative samples. The boundary samples, representative samples and equidistantly sampled normal samples were used together as training samples and the rest as test samples. A total of 13 sets of training data and 7 sets of test data were selected, as shown in Table 6 and Table 7.

The NURBS curves with 20 control points are fitted with these 13 sets of training data, and the fitted curves are sampled at equal intervals and expanded to generate 200 sets of data from the 13 sets of sample data. Then, the expanded data are used to construct a self-constructed fuzzy network with the input value of strain, and the calibrated nodal DOF was output through the trained self-constructed fuzzy network. Finally, the calibrated displacement values are obtained according to Equation (2) based on the calibrated nodal DOFs and the shape function *H*(*x*). In order to verify the verification accuracy of the CFTSC, the calibration accuracy index is shown.

(45) $$\mathrm{Error}=\left|disp^{n}-disp^{cf}\right|$$

(46) $$\mathrm{MER}=\mathrm{MAX}\left|disp^{n}\left(i\right)-disp^{cf}\left(i\right)\right|$$

(47) $$\mathrm{RMSE}=\sqrt{\frac{{\sum_{i=1}^{N}\left(disp^{n}\left(i\right)-disp^{cf}\left(i\right)\right)}^{2}}{N}}$$

where the superscript ‘*cf*’ indicates the CFTSC method. MR denotes the maximum displacement of the structure. The error was indicated by the absolute error, and MER indicates the maximum absolute error. When the load was at the maximum load state (400 N), the maximum displacement value in the z-direction of the main deformation direction was 158.83 mm.

**Figure 12:**
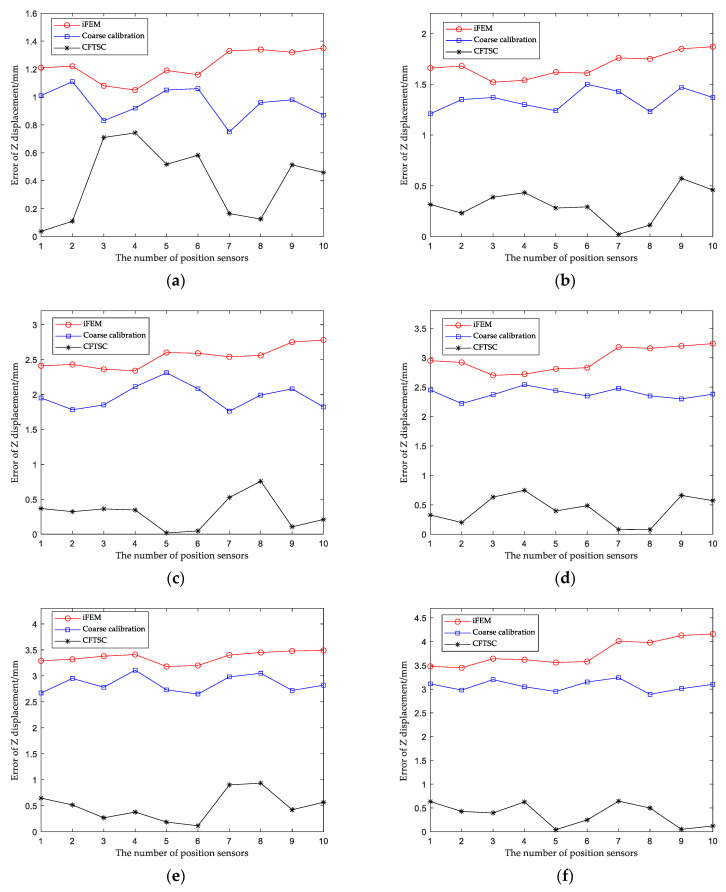

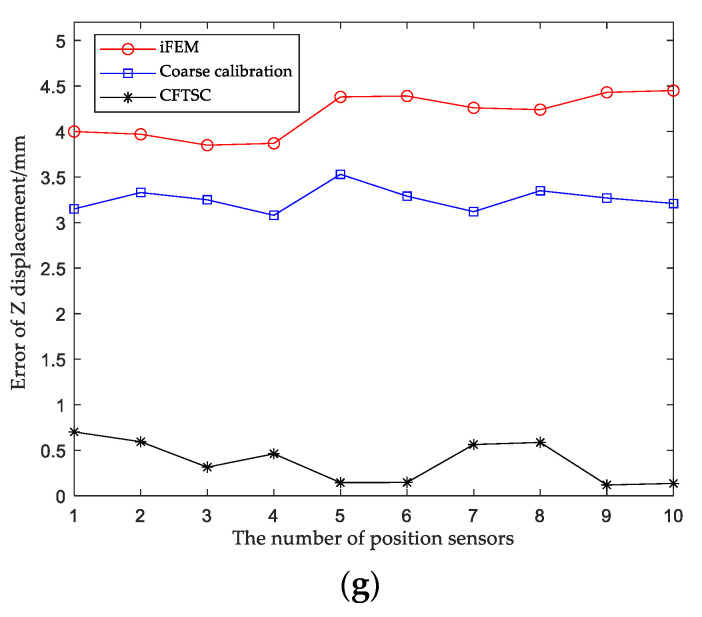
Comparison of CFTSC, Coarse calibration and iFEM displacements under test loads: (**a**) 80 N; (**b**) 120 N; (**c**) 200 N; (**d**) 240 N; (**e**)280 N; (**f**) 320 N; (**g**) 360 N.

**Figure 13:**
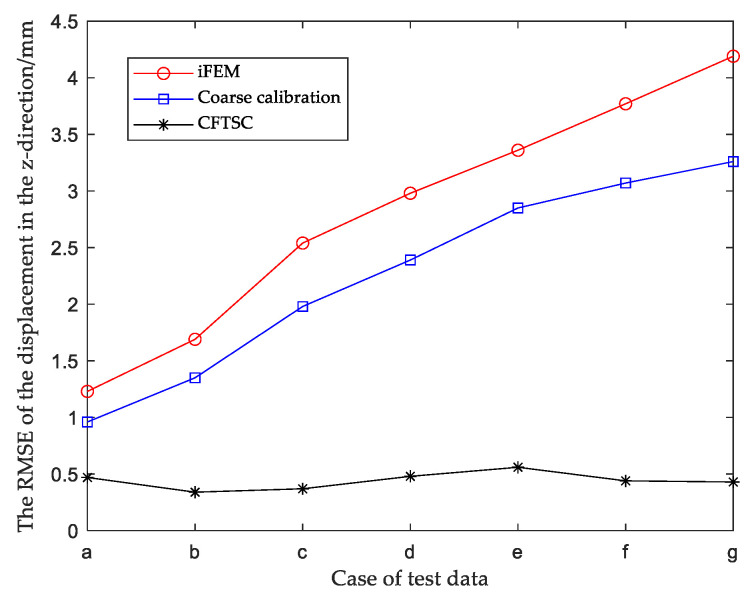
Comparison of RMSE under test data for CFTSC, Coarse calibration and iFEM.

**Figure 14:**
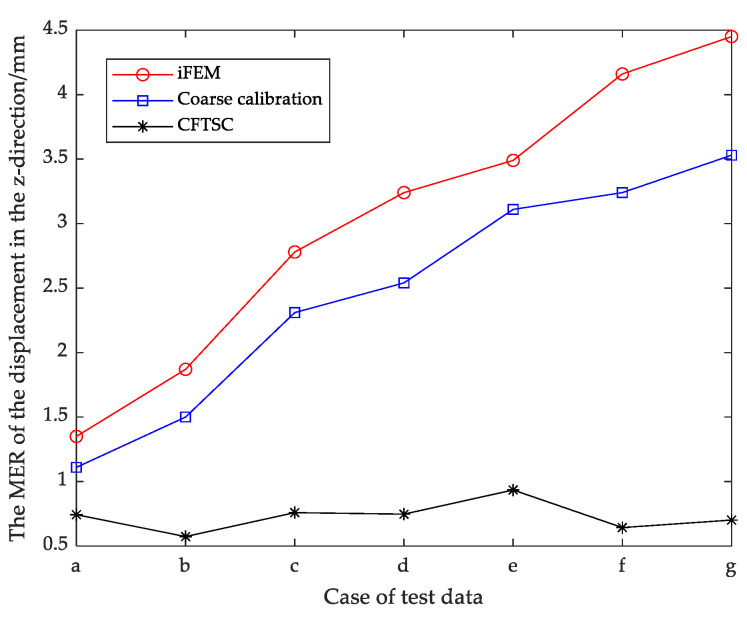
Comparison of MER under test data for CFTSC, Coarse calibration and iFEM.

The calibration effect of CFTSC was tested with seven sets of screened test data, and the calibration results of each set of test data are shown in Figure 12a–g, Figure 13 and Figure 14. The experimental results show that the displacement accuracy of the seven sets of test data was greatly improved compared with the traditional iFEM.

The reconstruction displacement error in the z-direction at the check point position can be obtained from Table 8. When the structure was under 360 N bending load, MR = 146.06 mm, the reconstructed displacement error MER = 3.53 mm and RMSE = 3.26 mm after coarse calibration. The reconstructed displacement error MER = 0.70 mm and RMSE = 0.43 mm after CFTSC on the basis of coarse calibration, compared with MER = 4.45 mm and RMSE = 4.19 mm based on the iFEM. As a result, based on the experimental results, it can be concluded that the CFTSC method was very effective in predicting displacement accuracy.

## 5. Conclusions

The CFTSC method based on the iFEM for shape perception is proposed to improve the reconstruction accuracy. The method effectively reduces the error between the iFEM-reconstructed displacement and the actual displacement. First, a coarse calibration is performed, and the displacement–strain matrix is corrected based on SOPSO to reduce the reconstruction error caused by the sensor offset during installation. On the basis of the coarse calibration, fine calibration is performed to establish the displacement nodal DOFs model and obtain the calibration values of the nodal DOFs based on the Bayesian algorithm. After that, the training and test data are screened based on the residual values under each operating condition, the sample size of the training data is expanded by NURBS, and then the relationship between the strain and nodal DOFs calibration values is obtained after training the self-constructed fuzzy network. Finally, the effectiveness of the CFTSC method is demonstrated experimentally; when the loading weight is 360 N and the maximum displacement is −146.06 mm, the maximum reconstruction error based on CFTSC method is 0.70 mm. Thus, the CFTSC method greatly improves the reconstruction accuracy of the displacement field.

## Figures and Tables

**Table 1:** Position of strain sensors.

Axial Position *x_p_*	0.21 L	0.22 L	0.575 L	0.53 L	0.505 L	0.56 L
(*θ*, *β*)	(20, 0)	(140, 0)	(−110, 0)	(110, 0)	(40, 0)	(160, 45)

**Table 2:** Position of Position sensors.

Number	Position (mm)	Number	Position (mm)
1	(562.04, 183.12, −4.74)	6	(1257.65, −169.87, 0.81)
2	(563.78, −176.30, 0.85)	7	(1585.45, 181.97, −3.38)
3	(909.58, 183.32, −5.42)	8	(1584.57, −176.63, 2.96)
4	(911.10, −179.36, 0.25)	9	(1933.19, 185.66, −0.94)
5	(1259.48, 183.39, −5.17)	10	(1934.84, −174.40, 4.14)

**Table 3:** Load weight for all working conditions.

**Number**	**1**	**2**	**3**	**4**	**5**	**6**	**7**
Load (N)	20 N	40 N	60 N	80 N	100 N	120 N	140 N
**Number**	**8**	**9**	**10**	**11**	**12**	**13**	**14**
Load (N)	160 N	180 N	200 N	220 N	240 N	260 N	280 N
**Number**	**15**	**16**	**17**	**18**	**19**	**20**	
Load (N)	300 N	320 N	340 N	360 N	380 N	400 N	

**Table 4:** Material properties of the model.

Properties	Beam-Plate	Rib
Density	2712 kg/m^3^	7850 kg/m^3^
Elastic modulus	7300 Mpa	21,000 Mpa
Poisson	0.3	0.3

**Table 5:** RMSE of coarse calibration and IFEM or each working condition unit: millimeters.

**Load (N)**	**20 N**	**40 N**	**60 N**	**80 N**	**100 N**	**120 N**	**140 N**	**160 N**	**180 N**	**200 N**
RMSE*^m^*	0.87	0.95	1.10	1.23	1.46	1.69	1.94	2.06	2.29	2.54
RMSE*^c^*	0.67	0.78	0.89	0.96	1.18	1.35	1.58	1.67	1.85	1.98
**Load (N)**	**220 N**	**240 N**	**260 N**	**280 N**	**300 N**	**320 N**	**340 N**	**360 N**	**380 N**	**400 N**
RMSE*^m^*	2.73	2.98	3.15	3.36	3.52	3.77	3.91	4.19	4.31	4.55
RMSE*^c^*	2.13	2.39	2.68	2.85	2.99	3.07	3.13	3.26	3.37	3.58

**Table 6:** Load in different working conditions for training.

Number	1	2	3	5	7	8	9	11	13	15	17	19	20
Load (N)	20 N	40 N	60 N	100 N	140 N	160 N	180 N	220 N	260 N	300 N	340 N	380 N	400 N

**Table 7:** Load in different working conditions for testing.

Number	4	6	10	12	14	16	18
Load (N)	80 N	120 N	200 N	240 N	280 N	320 N	360 N

**Table 8:** Comparisons of Z-direction measured and computed displacement unit: millimeters.

Case	a	b	c	d	e	f	g
MR^n^	−68.36	−79.39	−102.38	−113.00	−123.56	−134.36	−146.06
MR*^cf^*	−67.90	−78.93	−102.73	−112.43	−124.13	−134.58	−146.20
MR*^c^*	−67.49	−78.02	−100.56	−110.62	−121.18	−131.26	−142.85
MR*^m^*	−67.01	−77.52	−99.60	−109.76	−120.07	−130.20	−141.61
MER*^cf^*	0.74	0.57	0.75	0.74	0.93	0.64	0.70
MER*^c^*	1.11	1.50	2.31	2.54	3.11	3.24	3.53
MER*^m^*	1.35	1.87	2.78	3.24	3.49	4.16	4.45
RMSE*^cf^*	0.47	0.35	0.37	0.48	0.56	0.44	0.43
RMSE*^c^*	0.96	1.35	1.98	2.39	2.85	3.07	3.26
RMSE*^m^*	1.23	1.69	2.54	2.98	3.36	3.77	4.19

## Data Availability

Not applicable.

## Abbreviations

iFEM	Inverse finite element method
CFTSC	Coarse and fine two-stage calibration
SCFN	Self-constructed fuzzy networks
PSO	Swarm optimization algorithm
SOPSO	Single-objective particle swarm optimization algorithm
NURBS	Non Uniform Rational B-spline
DOF	Degrees of freedom
MCMC	Markov Chain Monte Carlo
RMSE	Root mean square error
RRMSE	Relative root mean square error
NDI	Northern digital incorporated
FBG	Fiber bragg grating
**List of Symbols**	
e(u)	Theoretical sectional strain
$e^{\varepsilon }$	Actual sectional strain
$d^{t}$	The displacement at any position of the section
H(x)	Shape function
$u_{e}$	Nodal degrees of freedom
$e_{u}$	The strain at any position of the cross-section
*M*(*x*)	Strain function matrix
$k^{e}$	Elements stiffness matrix
$f^{\mu }$	Elements stiffness vector
*l*	The unit length after reconstruction
*n*	The number of section strains on the neutral axis
$x_{i}$	The location of the calculated section strain.
$w_{p}$	Weighting factor
$\varepsilon^{a}$	Surface strains
$x_{i}$	Sensor axial coordinates
$\theta_{i}$	Sensor circumference angle
$\beta_{i}$	Angle between sensor direction and axial direction
T	Conversion relationship between surface strain and section strain
μ	Poisson ratio
r	The outer radius of the section
$D_{y}$	Bending stiffness in y-direction
$G_{z}$	Shear stiffness in z-direction
$T_{k}$	Displacement–strain transformation matrix
*u*,*v*,*x*	Displacement along each axis
*θ_x_*, *θ_y_*, *θ_z_*	Rotation angles of each axis
d	The displacement of the final reconstruction
$T_{k}^{\prime }$	Actual displacement–strain transformation matrix
$disp_{i}^{u}$	actual measured displacement at check points
$disp_{i}^{v}$	Theoretical displacements reconstructed by iFEM
*n*	The number of position sensors (check points)
*P*′	The actual installation positions of strain sensors
$d_{i}^{t}$	The theoretical displacement of the iFEM reconstruction
$d_{i}^{a}$	Actual displacement
$\Delta d_{i}$	Reconstruction error
G	Displacement field matrix
$N_{i}$	Shape function
Δu	Nodal degrees of freedom error
β	The residuals after displacement error correction
$p(\Delta u,\sigma^{2})$	Joint prior distribution
*N*(Δ*u_j_*_,_$\Delta s_{j}^{2}$)	The conditional posterior distribution of Δ*u*
$Q_{j}$	The *j*th sample data under working conditions
$\varepsilon_{l}$	The *l*th strain value measured by the sensor
$\Delta u_{i}$	The kinematic variable in the nodal DOF error
*F_i_*	Decompose the original sample data *Q_j_*
$k_{i}$	Weight factors
$M_{i}$	Control vertex
$E_{i,c}(t)$	The *c*-th order B-spline basis function
$t_{i}$	knots
*T*	Nodal vector
$\hat{F}$ *_i_*	Fitted data
${\hat{\varepsilon }}_{l}$	Fitted strain
$\Delta {\hat{u}}_{i}$	Fitted kinematic variables
*a_i_*	The weighting constants corresponding to the kinematic variable error
$H_{ki}$	Transformation matrix
*e^t^*	The integrated residual value of sample under one working condition
$F^{sc}$	Screened data set
$S^{n}$	The nth fuzzy rule
$\varepsilon_{k}$	Input values for fuzzy self-configured networks
${\hat{p}}^{n}$	Output of the nth fuzzy rule
$w^{n}$	The value corresponding to fuzzy rule output
$q_{n}$	The weighting constants of the rule
$\eta_{t}$	The error standard of SCFN
*η_a_*	Predetermined error thresholds
$\hat{U}(c)$	Actual output of SCFN
U(c)	Desired output of SCFN
$R_{k}^{n}$	Membership degree
$a^{n}(i)$	The rule adjustment result at moment *i*
*V*	Adjust the adaptive speed during rule adjustment
$\varphi^{n}(i-1)$	The weighting constants of the nth rule at moment *i −* 1
